# Substrate Bias-Driven Structural and Mechanical Evolution of AlCrN and AlCrSiN Coatings via Reactive Magnetron Sputtering

**DOI:** 10.3390/ma18071671

**Published:** 2025-04-05

**Authors:** Du-Cheng Tsai, Rong-Hsin Huang, Zue-Chin Chang, Erh-Chiang Chen, Yen-Lin Huang, Fuh-Sheng Shieu

**Affiliations:** 1 Department of Materials Science and Engineering, National Chung Hsing University, Taichung 402202, Taiwan; 2 Department of Electrical Engineering, Da-Yeh University, Changhua 51591, Taiwan; 3 Department of Mechanical Engineering, National Chin-Yi University of Technology, Taichung 411030, Taiwan; 4 Metal Industries Research and Development Centre, Kaohsiung 811225, Taiwan

**Keywords:** AlCrN coating, AlCrSiN coating, magnetron sputtering, multi-element nitride, hard coating

## Abstract

AlCrN and AlCrSiN coatings were deposited via reactive magnetron sputtering. This study investigates the effects of radio frequency (RF) substrate bias, ranging from 0 V to 200 V, on the chemical composition, microstructure, and mechanical properties of the coatings. All crystalline coatings exhibited a single wurtzite-type hexagonal close-packed (hcp) structure. At a 0 V substrate bias, the AlCrN coating consisted of porous V-shaped columnar crystallites, while the AlCrSiN coating exhibited a porous, fiber-like amorphous structure. As the substrate bias increased, crystal growth was promoted, void density decreased, and the surface morphology transitioned from a textured to a more rounded appearance. Additionally, the preferred orientation shifted toward the (101) direction. However, at excessively high substrate bias, re-nucleation occurred, leading to grain refinement and increased film densification, which in turn caused a further shift in the preferred orientation toward the (002) plane. Due to its multi-element composition and the low solubility of Si in nitrides, AlCrSiN coatings tend to exhibit an amorphous growth tendency during sputtering. As a result, their microstructure is more sensitive to substrate bias. This sensitivity results in the formation of a highly dense structure with an optimal crystallite size at a substrate bias of 100 V, leading to a hardness of 22.6 GPa—surpassing that of the AlCrN coating.

## 1. Introduction

To meet the requirements of efficient production, it is essential to ensure that mechanical equipment and parts have high reliability and a long service life during their operational period. In actual use, mechanical parts are often subjected to harsh conditions, such as high temperatures, high speeds, and corrosive environments. The surface quality of mechanical parts directly affects their performance and lifespan, and local surface damage often leads to the failure of the entire part. Improving the wear resistance and corrosion resistance of part surfaces can save societal resources and reduce production costs. Applying coatings to the surface of mechanical parts is a common method for extending their service life.

Al_2_O_3_ is well known for its excellent thermal hardness, chemical inertness, and baseline oxidation resistance; however, due to its predominantly ionic bonds—where aluminum atoms donate electrons to oxygen atoms, creating a strong attraction between oppositely charged ions—the material exhibits weaker interfacial adhesion when applied as a coating to non-ceramic surfaces, leading to reduced mechanical adhesion to the substrate and increased likelihood of delamination under stress. TiAlN coatings build upon this foundation with enhanced oxidation protection: at high temperatures, TiAlN forms a protective Al_2_O_3_ thin film through selective oxidation, while the substitution of aluminum for titanium causes lattice distortion that introduces compressive stress, improving hardness through solid solution strengthening [1,2,3]. Further advancing oxidation resistance, AlTiN coatings incorporate higher aluminum content for superior protection, while the most advanced AlCrN coatings—which replace titanium with chromium—achieve exceptional oxidation resistance through the formation of both Al_2_O_3_ and Cr_2_O_3_ microstructures that create a dense, adherent passive film for synergistic surface protection.

The crystal structure and properties of AlCrN coatings are closely related to the Al content. The crystal structure of CrN is a NaCl-type structure, and when forming AlCrN, Al atoms are solid-soluted into the CrN crystal by replacing some of the Cr atoms. As the Al content increases, the crystal structure transitions from face-centered cubic to hexagonal. The XRD analysis results of Cr_1-x_Al_x_N (where x represents the atomic ratio) coatings prepared by arc ion plating showed that when x ≤ 0.6, the structure remains cubic [4]. In this range, Al atoms replace Cr atoms in the face-centered cubic CrN crystal, causing lattice distortion. This lattice distortion enhances residual compressive stress in the cubic phase, contributing to improved hardness and wear resistance. When x ≥ 0.7, the structure transitions to a hexagonal configuration, where Cr atoms replace Al atoms in the hexagonal AlN crystal, leading to lattice distortion. The critical Al content for the structural transition of AlCrN coatings varies according to the parameters and deposition technique. Due to the greater stability of h-AlN, a persistent driving force exists for the transformation of the cubic phase into the hexagonal phase. Therefore, at high temperatures with enough diffusion energy, AlCrN easily shifts to the hexagonal phase. The cubic AlCrN phase with high Al content is particularly noted for its high hardness, wear resistance, and excellent oxidation resistance, making it ideal for high-temperature applications [5]. However, during use, the cubic phase may transform into an hcp structure, potentially reducing its desirable properties [6,7]. This degradation is not merely due to the phase transformation itself but is primarily attributed to the stress generated by changes in molar volume during the phase transition. These stresses can induce cracking within the film and promote the formation of voids, which further compromise the coating’s mechanical and protective performance.

Enhancing the properties of CrAlN coatings can be achieved through the incorporation of a fourth alloying element. Among various candidates, Si has been identified as the most promising element for improving the mechanical properties and oxidation resistance of CrAlN coatings. In CrAlSiN coatings, the presence of Si can occur in two primary forms: (1) as a substitutional solid solution where Si atoms replace Cr or Al atoms within the CrAlN crystal lattice, or (2) as amorphous Si–N phases segregated at the grain boundaries of CrAlN. The addition of Si refines the microstructure, promoting a finer grain size that enhances hardness and wear resistance. Furthermore, the incorporation of Si improves plastic deformation resistance, oxidation resistance, and thermal stability by forming a protective amorphous Si–N barrier that inhibits grain coarsening and oxidation at elevated temperatures [8,9,10,11,12,13]. These combined effects make Si an essential alloying element for advancing the performance of CrAlN coatings in high-temperature and demanding applications.

Most studies have focused on the cubic CrAlN phase, while the formation and properties of the hcp phase remain relatively underexplored. The same applies to CrAlSiN coatings. One significant reason for this is that the mechanical properties of hcp-AlN are generally considered inferior to those of cubic CrN [7]. However, in applications requiring high Al content, hcp-CrAlN and hcp-CrAlSiN may present more suitable material choices under specific conditions. It is well known that the Al_2_O_3_ phase is more thermally stable and protective than the Cr_2_O_3_ phase at high temperatures [14]. The incorporation of high Al content into AlCrN can significantly enhance thermal stability and oxidation resistance. Zhang et al. [15]. conducted ab initio calculations to evaluate the phase stabilities of the fcc-NaCl and hcp-wurtzite ZnS structures of CrAlN, confirming that the hcp structure became more stable with higher Al content. Furthermore, Polcar et al. [11] observed that even with the presence of an hcp phase in AlCrSiN coatings, the oxidation resistance remained excellent and, in certain cases, was even further enhanced. This finding indicates that the hcp phase might offer specific advantages under certain high-temperature conditions, particularly in coatings with high Al content. However, further studies are needed to evaluate the properties of hcp-CrAlN and hcp-CrAlSiN coatings to fully understand their suitability for advanced coating applications, with the primary focus on assessing their structural and mechanical characteristics. Therefore, in this study, hcp-AlCrN thin films were prepared using an Al_70_Cr_30_ and Al_60_Cr_30_Si_10_ target via a magnetron sputtering system. Given that substrate bias plays a crucial role in enhancing film density and refining microstructure, this research focuses on investigating the effects of substrate bias on the structural evolution and mechanical properties of the coatings. Since high-temperature hard coatings are not only essential for electrically conductive materials but also widely applied in ceramics and other non-conductive substrates, a radio frequency (RF) bias was employed instead of a direct current (DC) bias. This approach prevents charge accumulation and ensures stable ion bombardment during deposition. Furthermore, this study aims to elucidate the influence of substrate bias on the crystallographic orientation and surface morphology of the films, providing critical insights into optimizing deposition parameters to enhance coating performance.

## 2. Experimental Procedure

The AlCrN and AlCrSiN coatings were deposited on Si (100) wafers and quartz substrates using a DC magnetron sputtering system (self-assembled setup) with equimolar Al_70_Cr_30_ and Al_60_Cr_30_Si_10_ targets, each 75 mm in diameter. Prior to deposition, the substrates were thoroughly cleaned and rinsed with ethanol and distilled water in an ultrasonic bath. The sputtering chamber was evacuated to a base pressure of 2 × 10^−6^ torr using a turbo pump. The deposition of the coatings was conducted in an Ar+N_2_ mixed atmosphere under a DC power of 250 W and a working pressure of 3 × 10^−3^ torr. During the process, the Ar and N_2_ flow rates were maintained at 40 and 10 sccm, respectively. The substrate bias, which served as the primary control parameter, was varied between 0 and 200 V. This bias was induced by a RF bias voltage to influence ion energy during deposition. The distance between the substrate and the target was set at 90 mm, and no external substrate heating was applied during deposition. The deposition time was adjusted to achieve an approximate thickness of 500 nm for the unbiased AlCrN and AlCrSiN coatings. The deposition times for the unbiased AlCrN and AlCrSiN coatings were 45.2 min and 39.1 min, respectively. The coating area was 50 mm × 50 mm, and thickness uniformity was ensured within ±3%. Prior to deposition, the targets were presputtered with Ar to remove surface oxide layers, ensuring optimal deposition conditions.

The chemical composition and electronic states of the elements in the coatings were analyzed using electron spectroscopy for chemical analysis (ESCA; PHI 5000 VersaProbe, ULVAC-PHI, Inc., Chigasaki, Japan), with monochromatic Al K_α_ radiation (1486.6 eV). High-resolution spectra were acquired for the constitute elements with a pass energy of 58.7 eV. The instrument was calibrated using the C 1s peak at 284.8 eV as a reference for charge correction. To minimize charging effects, a charge neutralization system utilizing low-energy electrons was employed during the measurements. Prior to the ESCA analysis, the samples underwent Ar⁺ sputtering (1 keV) for 30 s to remove surface contamination while minimizing alterations of the intrinsic chemical states. To ensure measurement reliability, each sample was analyzed three times. The phase and crystal structure of the coatings were characterized by X-ray diffractometry (XRD; BRUKER D8 Discover, Bruker, Billerica, MA, USA), utilizing Cu Kα radiation. Morphology studies and thickness measurements were performed using field-emission scanning electron microscopy (FE-SEM; JEOL JSM-6700F, Tokyo, Japan). Microstructural analysis was carried out with high-resolution field-emission transmission electron microscopy (TEM; FEI E.O. Tecnai F20, EFI, Hillsboro, OR, USA). The hardness and elastic modulus of the coatings were evaluated using a nanoindentation tester (Hysitron, Minneapolis, MN, USA) by analyzing load versus displacement curves. Each sample was tested at least five times to ensure reliability and accuracy of the data.

## 3. Results and Discussion

**Figure 1:**
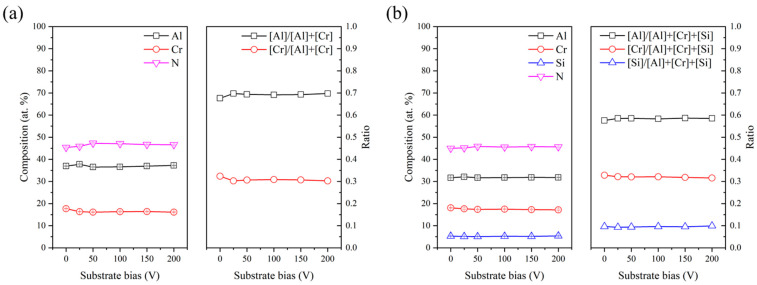
ESCA-determined composition as a function of substrate bias in the (**a**) AlCrN and (**b**) AlCrSiN coatings.

Figure 1 plots the chemical composition of the AlCrN and AlCrSiN coatings deposited at various substrate bias. The film composition exhibits negligible variation with increasing substrate bias, suggesting high compositional stability and uniformity across different bias conditions. This behavior is likely associated with the reaction stability of the nitride film during deposition and the consistent distribution of its constituents. Additionally, it is noted that the Al and Si contents in the coatings are slightly lower than those in the targets. These slight deviations from the target compositions are likely caused by variations in the sputtering yields of the constituent elements, as well as the effects of scattering and re-sputtering phenomena during the deposition process [16,17].

**Figure 2:**
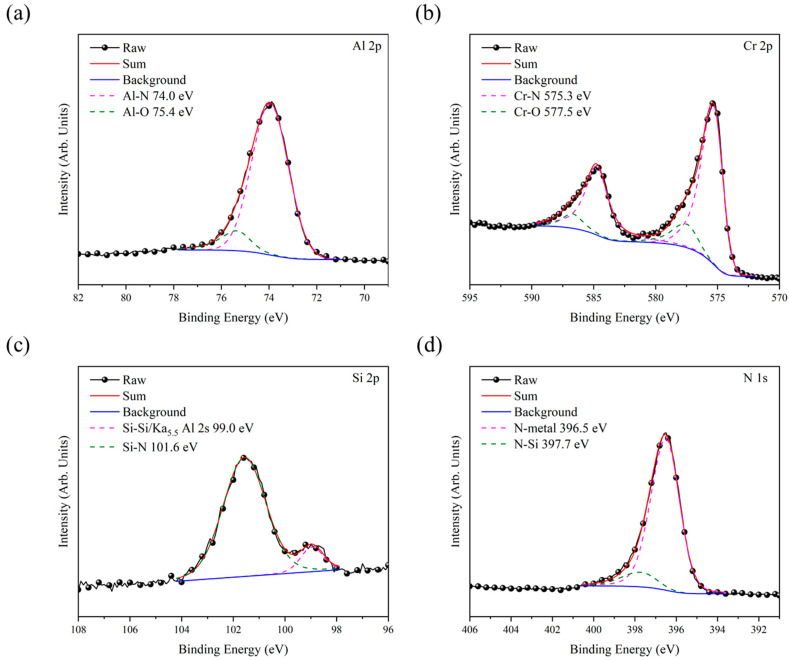
ESCA spectra of (**a**) Al 2p, (**b**) Cr 2p, (**c**) Si 2p, and (**d**) N 1 s for AlCrSiN coating.

Figure 2 presents the ESCA spectra of the Al 2s, Cr 2p, Si 2p, and N 1s core levels for the AlCrSiN coatings. In Figure 2a, the Al 2p spectrum is deconvoluted into two peaks located at 74.0 eV and 75.4 eV, corresponding to Al-N and Al-O bonds, respectively [18]. Figure 2b shows the Cr 2p spectrum, deconvoluted into two peaks at 575.3 eV and 577.5 eV, which are attributed to Cr-N and Cr-O bonds, respectively [19]. These results suggest that Al and Cr primarily exist as Al/Cr-N, with a small proportion in their oxidized states. The presence of strong Al/Cr-N bonds is critical for enhancing the mechanical strength of the coatings The existence of minor Al/Cr-O bonds suggests the partial oxidation of Al and Cr, possibly due to residual oxygen during deposition or post-exposure oxidation. Figure 2c depicts the Si 2p spectrum, with two deconvoluted peaks at 99.0 eV and 101.6 eV, which are assigned to Si-Si bonds or the Ka_5.5_ Al 2s plasmon and Si-N bonds in the Si_3_N_4_ phase, respectively [11]. Because the electronegativity of Si (1.90) is slightly higher than that of Al (1.61) and Cr (1.66), the electronegativity difference between Si and N is smaller, resulting in lower bond polarity. Consequently, the tendency to form Si-N bonds is weaker compared to Al-N and Cr-N bonds, leading to a lower proportion of Si-N bond formation. Lastly, Figure 2d shows the N 1s spectrum, deconvoluted into two peaks at 396.5 eV and 397.7 eV, which correspond to N-metal bonds and the N-Si plasmon, respectively [20]. These results indicate that Si doping primarily exists in the form of Si_3_N_4_, with a smaller fraction present as elemental Si within the AlCrN film, rather than being dissolved in the AlCrN lattice. Their incorporation into the AlCrN matrix disrupts the crystallization process by hindering atomic mobility and lattice ordering. As a result, the AlCrN film tends to exhibit increased amorphization, which may influence its mechanical properties.

**Figure 3:**
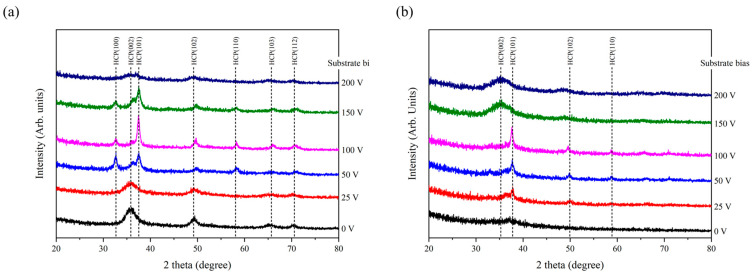
XRD patterns as a function of substrate bias in the (**a**) AlCrN and (**b**) AlCrSiN coatings.

Figure 3a,b present the XRD patterns of the AlCrN and AlCrSiN coatings deposited under various substrate bias conditions, respectively. To ensure accurate structural analysis, the coating with the highest crystallinity was selected for peak fitting. Specifically, the AlCrN coating deposited at a substrate bias of 100 V was analyzed for its structure, and the results are shown in Figure S1. The results of the analysis confirm a wurtzite-type hexagonal close-packed (hcp) structure with lattice constants a = 0.3163 nm and c = 0.4974 nm. Based on these findings, it can be concluded that all coatings exhibit a single-phase wurtzite-type hcp structure. As shown in Figure 3a, the diffraction peaks of the unbiased AlCrN coating are notably broad, indicating its poor crystallinity. Based on calculations using the Scherrer equation, the crystallite size is approximately 3.2 nm. Furthermore, the diffraction pattern reveals a (002) preferred orientation in the coating. Under deposition conditions without an applied bias, the adatom energy in the coating is relatively low, resulting in limited adatom mobility on the film surface. This restricted mobility inhibits the development of a well-ordered crystalline structure. The (002) plane, being thermodynamically stable and characterized by its low surface energy, is preferentially oriented during the deposition process [21]. The absence of substrate bias reduces the kinetic energy required for the formation of competing crystal orientations, thereby reinforcing the dominance of the (002) texture in AlCrN coatings. When the substrate bias is increased to 50 V, the diffraction peaks become sharper, indicating a significant improvement in the coating’s crystallinity. This improvement is accompanied by crystallite growth, with the crystallite size increasing to approximately 11.7 nm. Additionally, the (100) and (101) diffraction peaks exhibit nearly equivalent relative intensities. When the substrate bias reaches 100 V, the crystallite size of the coating reaches its maximum of 13.8 nm, and the preferred orientation changes to (101). During the sputtering process, the application of substrate bias induces high-energy ion bombardment on the substrate surface. Zhang et al. [22] synthesized CrN coatings using DC magnetron sputtering and analyzed the effects of different RF substrate biases on the deposition process using optical emission spectroscopy. The results showed that as the substrate bias increased, the ion flux near the substrate region increased, and the average ion energy incident on the growing film also rose. This bombardment transfers energy to the coating, increasing atom mobility and providing sufficient surface diffusion, thereby improving the crystallinity of the thin film. This effect is often described as a short-term localized annealing of the implantation-affected region, commonly referred to as a “thermal spike” [23,24]. On the other hand, high-energy ion bombardment can also cause damage to the film structure. In polycrystalline films, the sensitivity to such damage is strongly influenced by the orientation of the grains relative to the direction of ion incidence. For wurtzite-structured nitride, the most densely packed direction is the c-axis. Due to the tight atomic arrangement in this direction, the grains are more resistant to ion penetration. However, this dense structure also results in the localized concentration of ion energy, leading to more severe damage in specific regions. To mitigate such localized damage, the preferred orientation of the film tends to deviate from (002) [25]. In this study, the (101) plane was identified as the preferred orientation due to its lower atomic density, which facilitates better dispersion of ion energy and mitigates structural degradation, a characteristic feature of the ion channeling effect. With further increases in substrate bias to 200 V, the diffraction peaks become progressively weaker and eventually indistinguishable, indicating a substantial decline in the crystallinity of the thin film. The intensified ion bombardment at high substrate bias levels induces significant damage to the film during its growth, generating a high density of structural defects. These defects disrupt the atomic arrangement, impeding the formation of a well-ordered crystalline structure and promoting the transition toward an amorphous state [26,27,28]. However, the results of subsequent SEM and TEM analyses reveal that this is not the case. The “X-ray amorphous” characteristic does not necessarily indicate that the thin film structure is truly amorphous.

As shown in Figure 3b, the unbiased AlCrSiN coating exhibited almost no discernible diffraction peaks, indicating a structure that is nearly amorphous. Si atoms exhibited limited solubility in nitride crystalline phases and preferentially bonded with N atoms, resulting in the formation of an amorphous Si_3_N_4_ phase. This amorphous phase predominantly forms at the grain boundaries, where it effectively inhibits crystallization and grain growth [29]. As the bias voltage increased to 25 V, a sharp (101) diffraction peak emerged, indicating the onset of crystallization in the AlCrSiN coating. The average crystallite size of the (101)-oriented crystallites was about 8.0 nm. However, the presence of broad diffraction peaks strongly suggests that a substantial portion of the coating remained amorphous at this stage. When the bias voltage was further increased to 100 V, the diffraction peaks became progressively sharper, indicating enhanced crystallinity. Correspondingly, the crystallite size grew to approximately 11.5 nm, reflecting improved crystallization under the applied bias conditions. This change in preferred orientation can also be attributed to the same mechanism as in the AlCrN coatings, where ion channeling and reduced energy loss along the (101) direction, due to its more open structure over longer distances, play a dominant role. As the substrate bias continued to increase to 200 V, the diffraction peaks became very weak, similar to the behavior observed in the AlCrN coatings. This “X-ray amorphous” phenomenon appeared to occur readily under high bias conditions in this study. Although ion channeling initially favored the (101) orientation, excessive ion bombardment at high substrate bias voltage drastically reduced crystallite size. This led to a shift in the preferred orientation toward the lowest surface energy plane (200), overriding the ion channeling effect [30]. To clarify these structural changes, further investigation using SEM and TEM was conducted to provide deeper insights into the underlying microstructural features.

**Figure 4:**
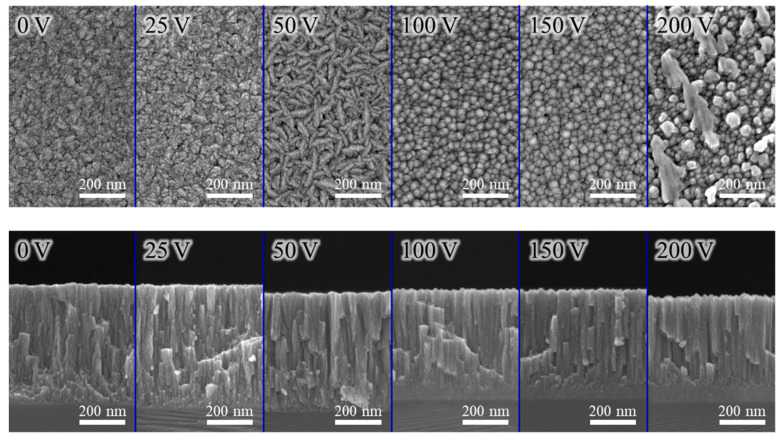
SEM images as a function of substrate bias in the AlCrN coatings.

**Figure 5:**
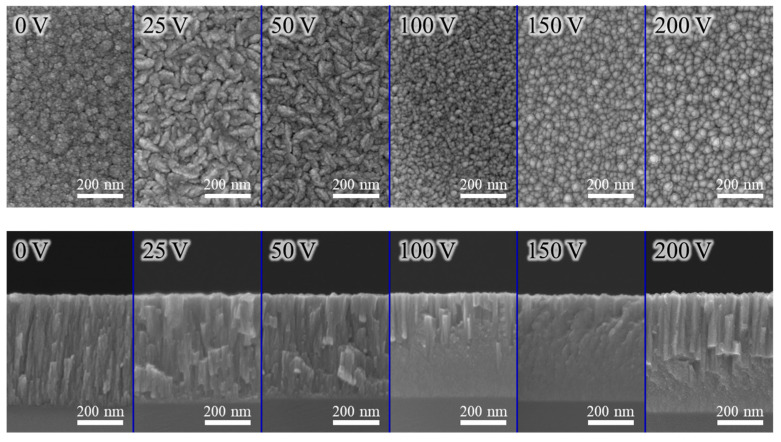
SEM images as a function of substrate bias in the AlCrSiN coatings.

Figure 4 and Figure 5 present typical SEM micrographs of the AlCrN and AlCrSiN coatings deposited under various substrate bias conditions, respectively. The coatings deposited without substrate bias exhibit a well-defined columnar structure with finely textured surface grains. As the substrate bias increased to 50 V, the enhanced atomic mobility facilitated grain growth within the coating, leading to the formation of larger and more prominent surface grains. At a substrate bias of 100 V, adatoms gained sufficient energy to overcome local energy barriers, allowing them to migrate more freely along the film surface. This increased mobility not only filled surface voids but also promoted atomic reorganization into a smoother morphology with rounded grains, reducing grain boundary irregularities and helping to minimize internal stress. Notably, for the AlCrN coating, when the substrate bias was further increased to 200 V, particles with abnormal size variations appeared on the coating surface. This phenomenon was likely caused by arc discharges induced by the excessively high bias voltage, which disrupted the uniform deposition process through localized overheating and material ejection. Moreover, these large particles were not uniformly distributed across the surface, and their sizes varied considerably, suggesting a non-uniform and sporadic nature of arc discharge events. However, from an overall perspective, the affected area remained relatively limited, with most particles only slightly larger than typical features. The coating uniformity can be assessed from lower-magnification SEM images, as shown in Figures S2 and S3, which demonstrate a high degree of uniformity across the deposited films.

**Figure 6:**
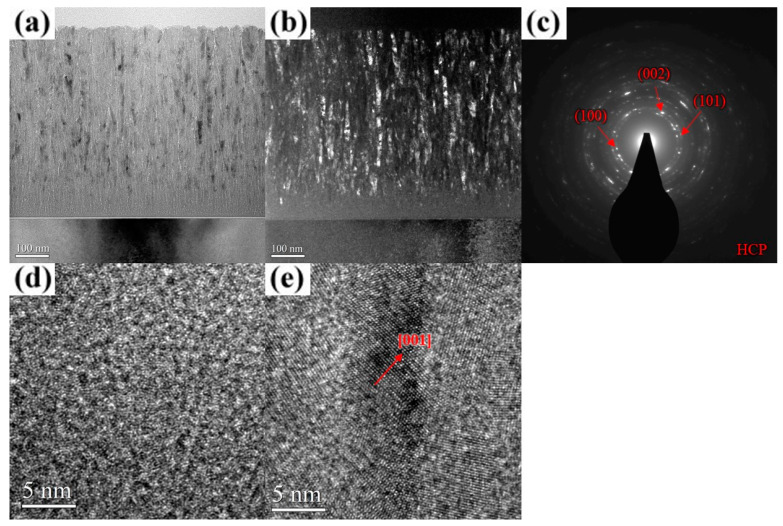
Cross-sectional TEM micrographs of the unbiased AlCrN coating. (**a**) Bright-field image. (**b**) Dark-field image. (**c**) SAD pattern. (**d**) HR-TEM image near the substrate. (**e**) HR-TEM image near the surface.

**Figure 7:**
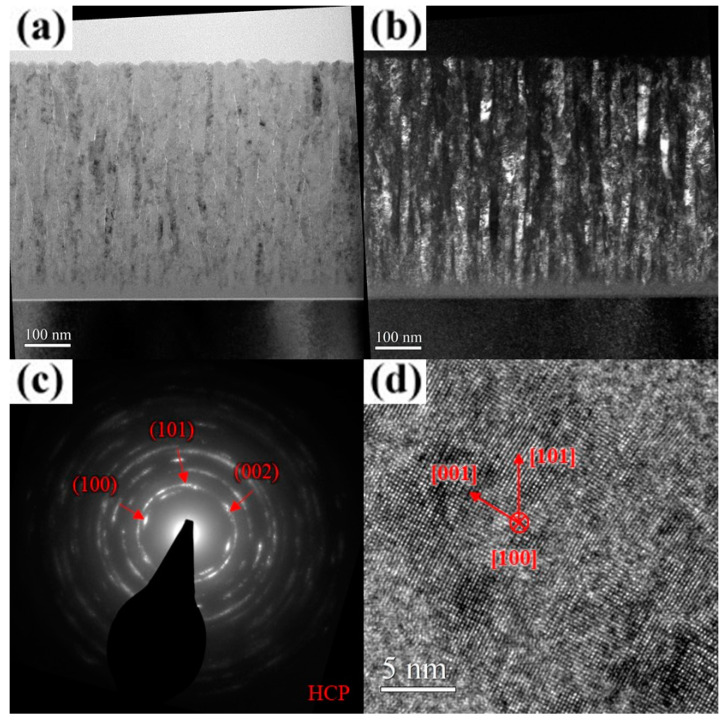
Cross-sectional TEM micrographs of the AlCrN coating deposited at a substrate bias of 100 V. (**a**) Bright-field image. (**b**) Dark-field image. (**c**) SAD pattern. (**d**) HR-TEM image near the surface.

**Figure 8:**
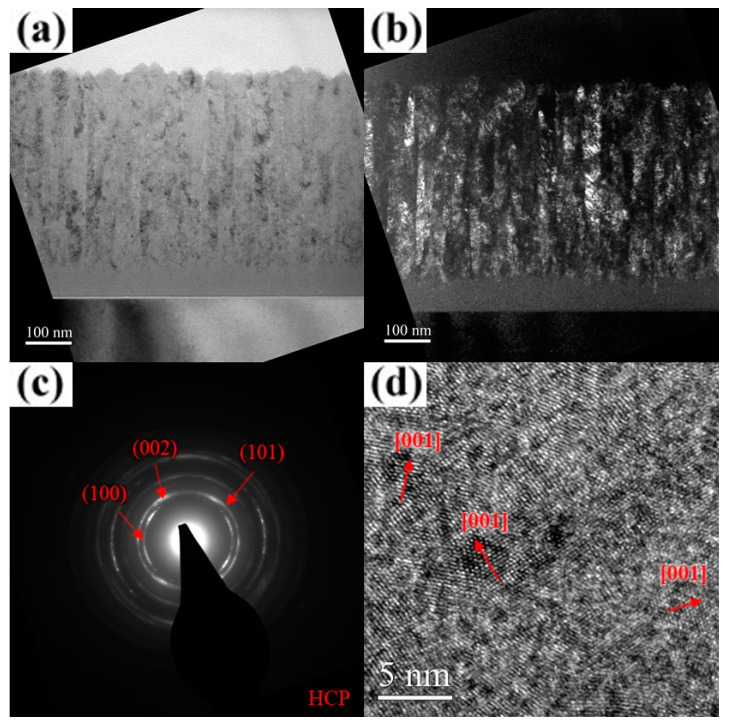
Cross-sectional TEM micrographs of the AlCrN coating deposited at a substrate bias of 200 V. (**a**) Bright-field image. (**b**) Dark-field image. (**c**) SAD pattern. (**d**) HR-TEM image near the surface.

**Figure 9:**
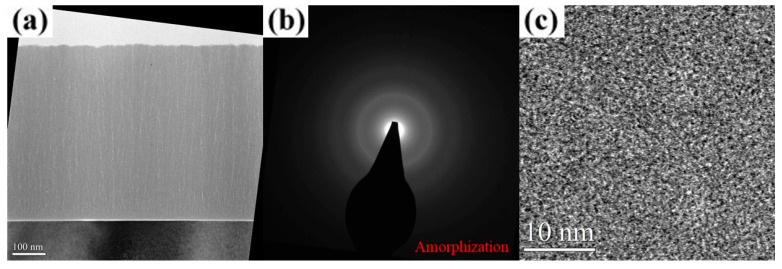
Cross-sectional TEM micrographs of the unbiased AlCrSiN coating. (**a**) Bright-field image. (**b**) SAD pattern. (**c**) HR-TEM image near the surface.

**Figure 10:**
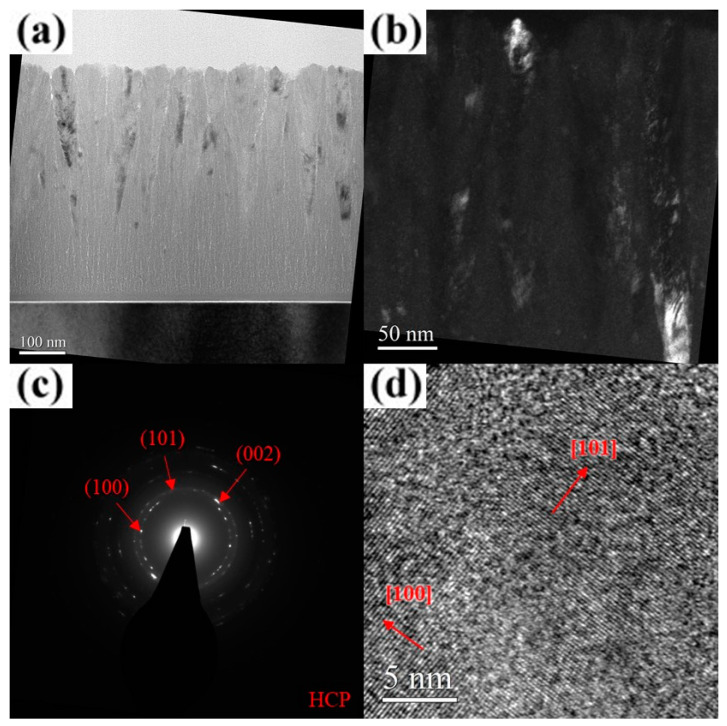
Cross-sectional TEM micrographs of the AlCrSiN coating deposited at a substrate bias of 25 V. (**a**) Bright-field image. (**b**) Dark-field image. (**c**) SAD pattern. (**d**) HR-TEM image near the surface.

**Figure 11:**
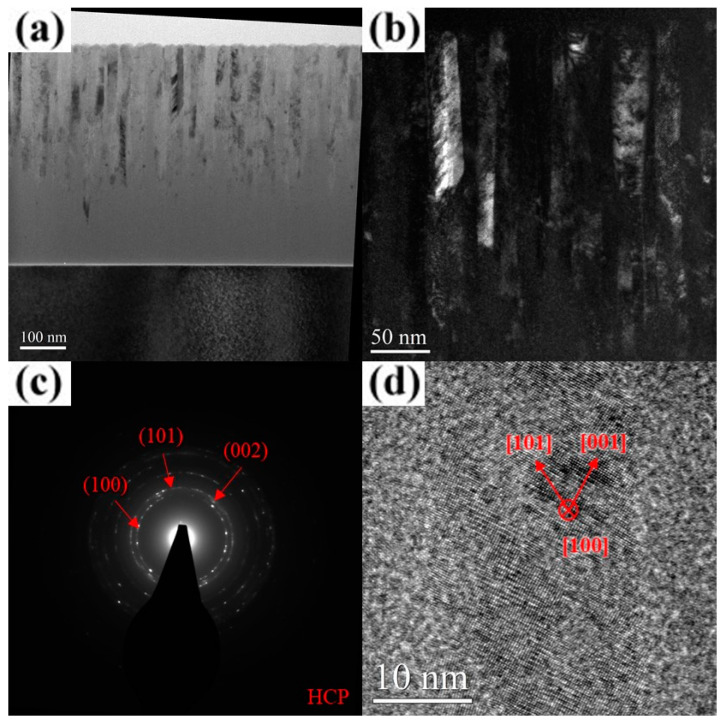
Cross-sectional TEM micrographs of the AlCrSiN coating deposited at a substrate bias of 100 V. (**a**) Bright-field image. (**b**) Dark-field image. (**c**) SAD pattern. (**d**) HR-TEM image near the surface.

**Figure 12:**
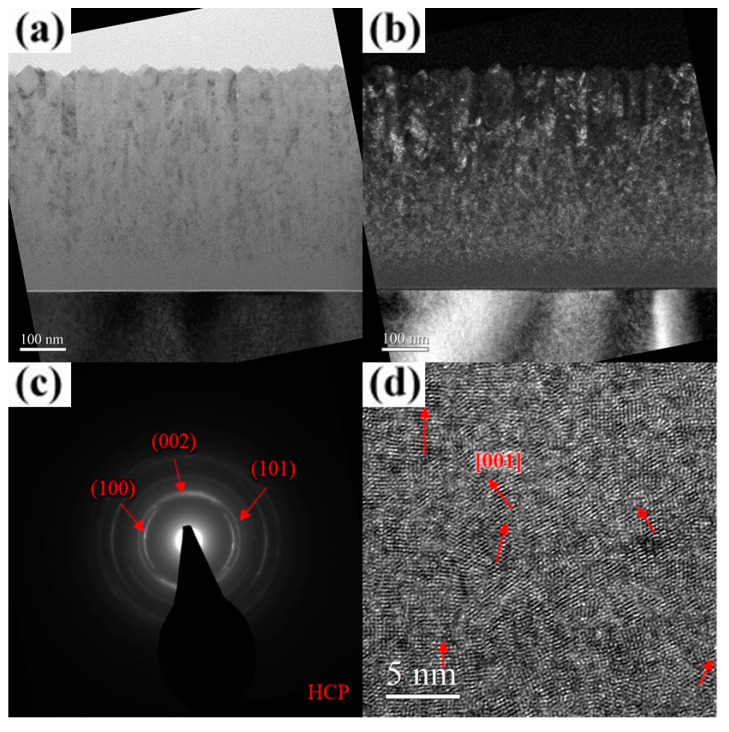
Cross-sectional TEM micrographs of the AlCrSiN coating deposited at a substrate bias of 200 V. (**a**) Bright-field image. (**b**) Dark-field image. (**c**) SAD pattern. (**d**) HR-TEM image near the surface.

The TEM images with selected-area diffraction (SAD) patterns of the AlCrN and AlCrSiN coatings deposited under various substrate bias conditions are presented in Figure 6, Figure 7, Figure 8, Figure 9, Figure 10, Figure 11 and Figure 12. As shown in Figure 6a, the unbiased AlCrN coating exhibited a porous V-shaped columnar structure, a morphology characteristic of an evolutionary overgrowth mechanism [31]. Significant intra- and inter-column porosities were observed, indicating a relatively low-density microstructure. Each columnar grain comprised multiple fibrous crystallites aligned along the film growth direction, with an average crystallite width of approximately 11 nm, as determined from the dark-field image (Figure 6b). The SAD pattern in Figure 6c, obtained using a 0.2 μm aperture, reveals diffraction segments corresponding to the (100), (002), and (101) planes of an hcp structure, confirming the presence of nanocrystalline phases within the coating. The results of high-resolution TEM (HR-TEM) analysis further reveal a structural gradient within the film. The region near the substrate exhibited an amorphous structure with a thickness of approximately 65 nm (Figure 6a,d), while the near-surface region consisted of crystallites with distinct hcp [001] lattice fringes (Figure 6e). With an increase in the substrate bias to 100 V, the coating structure underwent significant changes. Intra- and inter-column porosities were substantially reduced (Figure 7a), while the thickness of the amorphous layer near the substrate decreased to approximately 28 nm. The crystallite width, as observed in the dark-field images, increased to approximately 20–26 nm (Figure 7b). The SAD pattern and HR-TEM image further confirm that the coatings exhibited a well-ordered hcp crystal structure with a preferential (101) out-of-plane orientation (Figure 7c,d), indicating improved crystallinity under increased bias conditions. At a substrate bias of 200 V, the film retained its columnar morphology, with no significant change in column width, except for some surface undulations likely caused by arc discharges, while the thickness of the amorphous layer near the substrate increased to approximately 70 nm (Figure 8a). Additionally, intra- and inter-column porosities were further reduced compared to the 100 V condition, suggesting a denser microstructure. However, dark-field images and the SAD pattern (Figure 8b,c) indicate that the film still maintained good crystallinity, despite XRD analysis suggesting an almost entirely amorphous structure under this condition. A closer examination of the dark-field image reveals that, compared to the films deposited at 100 V, the crystallites in the 200 V film exhibited a less elongated and distinctly inclined columnar shape. Due to this inclined columnar structure, evaluating the crystallite width is challenging, but overall, the width is estimated to have decreased to approximately 8–12 nm. Additionally, the SAD pattern (Figure 8c) presents arc-shaped and slightly diffuse diffraction rings, indicating that the film consisted of fine crystallites with different orientations. The HR-TEM analysis results (Figure 8d) further corroborate the inconsistency in the preferred orientation of these crystallites, providing strong evidence that high substrate bias not only disrupted columnar crystallite growth but also induced crystallite refinement, leading to increased orientation dispersion within the film. These observations suggest that high bias voltage induced a certain degree of structural damage, promoting re-nucleation events that contributed to a reduction in crystallite size [32]. However, the primary impact appeared to be on the length of the elongated crystallites along the film growth direction, whereas the width was relatively less affected, ultimately contributing to the “X-ray amorphous” phenomenon. This “X-ray amorphous” phenomenon can be attributed to the misalignment of crystalline grains within the film. Specifically, films can contain grains where the planes of reflection are not parallel to the film–substrate interface, resulting in a larger deviation between the grain growth direction and the overall film growth direction [33,34]. Consequently, the reduced alignment of crystalline planes with the X-ray beam led to diminished diffraction peak intensity, creating the misleading appearance of an amorphous structure in XRD analysis.

As shown in Figure 9a, the unbiased AlCrSiN coating exhibits a porous fiber structure with no discernible diffraction contrast, indicating a lack of well-formed crystalline domains. The corresponding SAD pattern (Figure 9b) presents a uniform and diffuse halo ring, further confirming the absence of long-range crystallinity. Moreover, the HR-TEM image (Figure 9c) reveals an irregular “granular” or “hazy” contrast, rather than distinct lattice fringes. These observations collectively provide strong evidence that Si incorporation promoted amorphization within the coating. As the substrate bias increased to 25 V, the film underwent rapid surface crystallization, forming a V-shaped columnar structure through the evolutionary overgrowth mechanism, while the underlying region remained amorphous, with a thickness ranging from approximately 155 to 285 nm. (Figure 10a) The width of the crystallites near the surface, as observed in the dark-field images, ranged from approximately 15 to 19 nm (Figure 10b). The corresponding SAD pattern and HR-TEM image (Figure 10c,d) confirm that the coating adopted an hcp crystal structure with a preferential (101) out-of-plane orientation. A detailed examination of Figure 10a reveals that, due to the bias-induced crystallization of the amorphous surface layer, the inter-column voids widened rather than decreased. When an amorphous region transforms into a crystalline phase, the atomic arrangement becomes more compact compared to the disordered amorphous structure. This densification typically results in local volume contraction, which in turn causes the surrounding voids to appear wider relative to their initial state. A similar phenomenon was reported by Tsai et al., who observed that introducing nitrogen gas during the growth of (TiVCr)N films led to significant crystallite growth while simultaneously creating pronounced voids around the crystallites [35]. At a substrate bias of 100 V, crystallization within the film was further enhanced, leading to a reduction in the thickness of the underlying amorphous region to approximately 155–205 nm. The overall film structure became significantly denser, with nearly no visible voids (Figure 11a). The dark-field image reveals a substantial increase in the length of the elongated crystallites along the film growth direction (Figure 11b). The corresponding SAD pattern and HR-TEM image (Figure 11c,d) further confirm the improved crystallinity of the film, with a more pronounced preferential (101) out-of-plane orientation. For the AlCrSiN coating, the extent of crystallization under bias conditions was lower than that of the AlCrN coating, which aligns with expectations. The results of ESCA analysis indicate that Si did not exist in a solid solution form within AlCrN but rather as Si_3_N_4_ and elemental Si, which significantly hindered crystallite growth during deposition. Notably, the voids within the AlCrSiN film disappeared entirely, a phenomenon attributed to the influence of Si incorporation. The presence of Si promoted the formation of an amorphous matrix, which suppressed crystallite growth and thus facilitated densification. This effect was also observed by Shen and Tsai et al., where Si-containing nitride coatings exhibited significantly higher structural density compared to their Si-free counterparts [36,37,38]. As the substrate bias increased to 200 V, the columnar grain boundaries became less distinct (Figure 12a). The thickness of the amorphous layer near the substrate, although decreasing to approximately 55 nm, was accompanied by a significant deterioration in the crystalline quality of the overlying structure. The dark-field image reveals a significant reduction in crystallite size, which was considerably smaller than that observed in the AlCrN coating, exhibiting a transition toward equiaxed crystallite growth (Figure 12b). The SAD pattern (Figure 12c) closely resembles that of the AlCrN coating, displaying arc-shaped diffraction rings, but with weaker intensity and a more diffuse appearance. The observed out-of-plane orientation was predominantly along the (002) plane. Additionally, the HR-TEM analysis results (Figure 12d) further confirm that the crystallites were smaller and more randomly oriented compared to those in the AlCrN coating, with a crystallite width of approximately 2–5 nm. A comprehensive analysis of the observed phenomena revealed that the substrate bias had a more pronounced effect on the structure of the AlCrSiN coatings, which inherently exhibited lower crystallinity during growth. This influence was particularly evident in terms of crystallite size, morphology, and structure densification.

**Figure 13:**
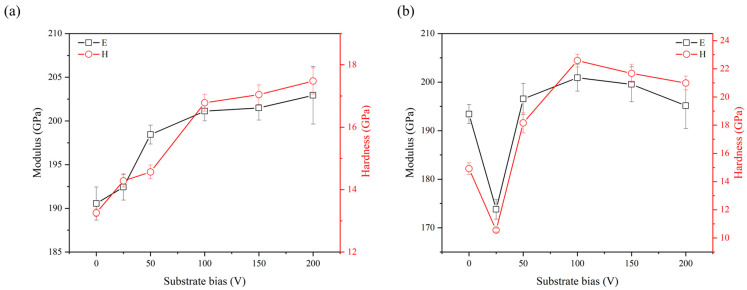
Hardness and elastic modulus as a function substrate bias in the (**a**) AlCrN and (**b**) AlCrSiN coatings.

The influence of substrate bias on the hardness and elastic modulus of the AlCrN and AlCrSiN coatings is illustrated in Figure 13a and b, respectively. As shown in Figure 13a, the hardness of the AlCrN coatings exhibited a monotonic increase from 13.2 GPa to 17.4 GPa with increasing substrate bias, while the elastic modulus correspondingly rose from 190.5 GPa to 202.9 GPa. In contrast, as shown in Figure 13b, the hardness and elastic modulus of the AlCrSiN coatings followed a non-monotonic trend. Initially, as the substrate bias increased to 25 V, the hardness and elastic modulus decreased from 14.9 GPa to 10.6 GPa and from 193.4 GPa to 173.8 GPa, respectively. However, with a further increase in bias to 100 V, both values reached their respective peak values of approximately 22.6 GPa and 200.9 GPa. Beyond this point, as the substrate bias increased to 200 V, the hardness and elastic modulus exhibited a slight decline to 21.0 GPa and 195.2 GPa, respectively. Based on the structural analysis presented earlier, it is evident that increasing the substrate bias significantly affected the crystallite size, structure densification, and preferential orientation of the coatings. In contrast, compositional variations were minimal and can be considered negligible. While some studies have suggested that preferential orientation influences the mechanical properties of coatings, particularly for hcp-AlN with a highly (002)-oriented plane, where atoms are arranged more closely in the direction perpendicular to the film surface, leading to stronger atomic bonding and enhanced interatomic forces [39], this reasoning does not align with the findings of the present study. Moreover, a larger body of literature suggests that the effect of preferential orientation on mechanical properties is relatively minor. Therefore, in this study, the observed trends in mechanical properties as a function of substrate bias can be primarily attributed to changes in crystallite size and structure densification. For the AlCrN coatings, the lowest hardness was observed in the unbiased coating, which can be attributed to its high porosity and extremely small crystallite size of 3.2 nm. According to the inverse Hall–Petch effect, when the crystallite size decreases below approximately 10 nm, dislocation-mediated plasticity becomes less dominant, and grain boundary sliding or atomic diffusion mechanisms govern deformation, leading to a reduction in hardness [40]. However, it is important to note that the onset of the inverse Hall–Petch effect does not have a universally defined crystallite size threshold, as it is influenced by factors such as material composition, grain boundary structure, and defect density [41,42]. With an increase in substrate bias to 100 V, the coating underwent significant structural modifications, leading to an increase in both film densification and crystallite size to 13.8 nm. As a result, the hardness improved considerably. However, as the bias was further increased to 200 V, the hardness exhibited only a marginal increase. This limited hardness enhancement can be attributed to the combined effects of a slight increase in film density and a reduction in crystallite size, as further supported by HR-TEM dark-field imaging, which revealed crystallite sizes of approximately 8–12 nm. Such fine crystallite sizes may have triggered the inverse Hall–Petch effect, thereby counteracting the expected hardness improvement.

For the AlCrSiN coatings, the unbiased coating exhibited relatively low hardness due to its porous amorphous structure. When the substrate bias increased to 25 V, the hardness decreased significantly, which can be attributed to the crystallization-induced widening of voids [35]. As the substrate bias further increased to 100 V, the hardness rose rapidly, coinciding with a highly densified film structure where no visible voids were present. However, as the substrate bias increased to 200 V, HR-TEM imaging reveals a reduction in crystallite size to 2–5 nm, which triggered the inverse Hall–Petch effect, becoming the dominant factor that led to a decrease in hardness. At this stage, while the film remained highly dense, the refinement of crystallites reduced the effectiveness of dislocation-mediated strengthening mechanisms. Instead, grain boundary sliding and atomic diffusion may have contributed to localized plastic deformation, resulting in a slight decrease in hardness [43]. As observed earlier, the incorporation of Si into the coating had a significant influence on its structural evolution under increasing substrate bias, which in turn affected its mechanical properties. Overall, the AlCrSiN coatings exhibited higher hardness than the AlCrN coatings across the range of substrate bias conditions. The highest hardness was observed at a substrate bias of 100 V for the AlCrSiN coating, which can be attributed to its highly densified microstructure and optimal crystallite size. This structure enhanced dislocation-mediated strengthening while minimizing the adverse effects associated with excessive grain refinement, resulting in superior mechanical performance.

The experimental results indicate the presence of two distinct regions regarding the effect of substrate bias on the coating structure, suggesting that different mechanisms dominated the deposition process across different substrate bias ranges. At low substrate bias, an increase in bias voltage induced four key effects: (i) crystallite growth, (ii) the development of (101) preferred orientation, (iii) a more rounded surface morphology, and (iv) structural densification. In the absence of an applied bias voltage, the coatings exhibited high porosity and poor crystallinity, with a textured surface grain morphology. When a substrate bias was introduced, the gas and metallic ions, accelerated by the substrate voltage, bombarded the growing film. This process simultaneously enhanced adatom mobility and induced structural damage. Given that (101) crystallites feature open channel directions, they can withstand high-energy ion irradiation with minimal lattice distortion. As a result, the film underwent a transition toward a denser columnar structure, characterized by a more rounded surface morphology and the growth of (101)-oriented crystallites. At high substrate bias, the increase in bias voltage instead led to crystallite refinement and the transition of a (200) preferred orientation. Excessive bias voltage disrupted the structural integrity, initiating re-nucleation mechanisms that contributed to the decrease in crystallite size. As a consequence, the ion channeling effect was weakened, favoring the development of a (002) preferred orientation, which corresponds to the lowest surface energy plane. This shift in orientation suggests that, under extreme bias conditions, the coating structure underwent a stabilization mechanism, where atomic rearrangement was driven by energy minimization rather than by lattice distortion tolerance. A comparison between the AlCrN and AlCrSiN coatings revealed that the AlCrSiN exhibited lower crystallinity than the AlCrN coatings but achieved higher structure densification. The reduced crystallinity is attributed to the presence of amorphous Si_3_N_4_ and elemental Si, which suppressed crystallite growth. The higher structure densification was due to the more random atomic arrangement of low-crystallinity films, which enabled a more uniform filling of different regions, minimizing voids caused by crystalline arrangement constraints and thereby resulting in a more compact structure compared to highly crystalline films. However, an exception was observed in the case of the unbiased AlCrSiN coating, which initially exhibited an amorphous structure. Under a substrate bias of 25 V, rapid surface crystallization occurred, leading to an unexpected effect—instead of structure densification, the rapid formation of crystallites induced localized volume contraction, generating significant internal stress within the material. As the accumulated stress reached a critical threshold, localized stress relaxation was triggered, ultimately causing a widening of inter-column voids. This phenomenon highlights the distinct response of the AlCrSiN coatings to bias-induced crystallization compared to AlCrN. These structural modifications significantly influenced the mechanical properties of the coatings. Based on the above analysis, an increase in substrate bias generally enhanced film densification, which was typically accompanied by an increase in hardness. The AlCrN coating exhibited a good correlation between densification and hardness enhancement, faithfully following this expected trend. However, in the case of the AlCrSiN coating, this relationship deviated, indicating that additional factors played a critical role in determining its mechanical behavior. For the AlCrSiN coating deposited at a substrate bias of 25 V, the widening of the inter-column voids resulted in poor hardness performance. However, as the substrate bias further increased, a significant structural transformation occurred, leading to the elimination of voids, improved densification, and a corresponding recovery in hardness. Nevertheless, when the substrate bias became excessively high, HRTEM observations revealed that the crystallite size was reduced to 2–5 nm, a range reported in the literature to trigger the inverse Hall–Petch effect. In this regime, the hardness decreased despite the maintained high film densification. These results suggest that an optimal bias range exists, balancing crystallinity, densification, and mechanical performance.

## 4. Conclusions

The AlCrN and AlCrSiN coatings prepared by reactive magnetron sputtering under different RF substrate bias conditions exhibited a single wurtzite-type hexagonal close-packed (hcp) structure. The unbiased AlCrN coating exhibited a porous V-shaped columnar structure with a textured surface, primarily due to the low adatom mobility during deposition. This porous microstructure contributed to its relatively low hardness of 13.2 GPa. With increasing substrate bias, ion bombardment enhanced adatom mobility, promoting a transition toward a denser columnar structure with a more rounded surface and [101]-oriented crystallite growth. However, residual porosity remained within the coating. As the substrate bias was further increased, intense ion bombardment induced grain refinement and a shift in the preferred orientation toward the (002) plane. Despite these structural modifications, inter-column porosities persisted, resulting in only a marginal improvement in hardness, reaching 17.4 GPa. These findings suggest that while higher bias facilitated structural densification, the persistence of residual porosity limited the extent of hardness enhancement. The unbiased AlCrSiN coating exhibited a porous, fiber-like amorphous structure with an initial hardness of 14.9 GPa. As the substrate bias increased to 25 V, the crystallization of the amorphous phase led to the widening of inter-column voids, resulting in a counterintuitive decrease in hardness to 10.6 GPa. With further increases in bias, the coating structure gradually became more compact, accompanied by the progressive growth of [101]-oriented crystallites. At a substrate bias of 100 V, the coating achieved a highly dense microstructure with an optimal crystallite size, leading to the highest hardness of 22.6 GPa. However, further increasing the substrate bias to 200 V induced excessive grain refinement, which triggered the inverse Hall–Petch effect, leading to a slight decrease in hardness to 21.0 GPa. This suggests that at excessively high bias, while densification is maintained, the reduction in crystallite size diminishes the effectiveness of dislocation-mediated strengthening mechanisms, ultimately limiting further improvements in hardness.

## Data Availability

Data are contained within this article.

## Supplementary Materials

The following supporting information can be downloaded at https://www.mdpi.com/article/10.3390/ma18071671/s1: Figure S1: The theoretical XRD pattern obtained through diffraction peak fitting of the AlCrN coating deposited at a substrate bias of 100 V; Figure S2: Low magnification SEM images as a function of substrate bias in the AlCrN coatings; Figure S3: Low magnification SEM images as a function of substrate bias in the AlCrSiN coatings.
