# Supplementary material for: Substrate Bias-Driven Structural and Mechanical Evolution of AlCrN and AlCrSiN Coatings via Reactive Magnetron Sputtering
Source: Materials (Basel). 2025 Apr 5;18(7):1671. doi: 10.3390/ma18071671 (PMC11990696; doi:10.3390/ma18071671)
Supplement: Supplementary file 1 [file materials-18-01671-s001.zip › materials-3500731-supplementary.pdf]

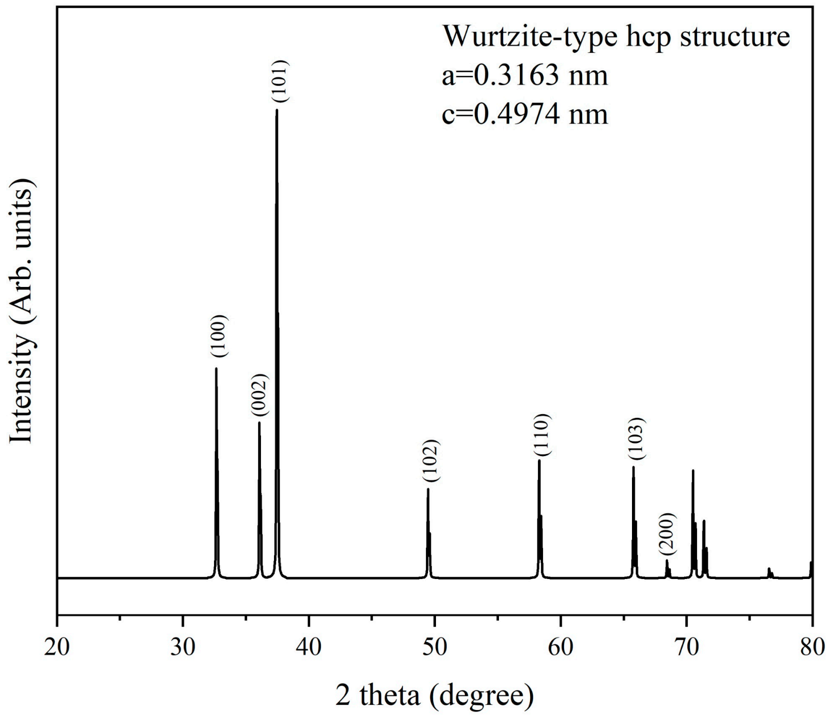

Figure S1. The theoretical XRD pattern obtained through diffraction peak fitting of the AlCrN coating deposited at a substrate bias of 100 V.

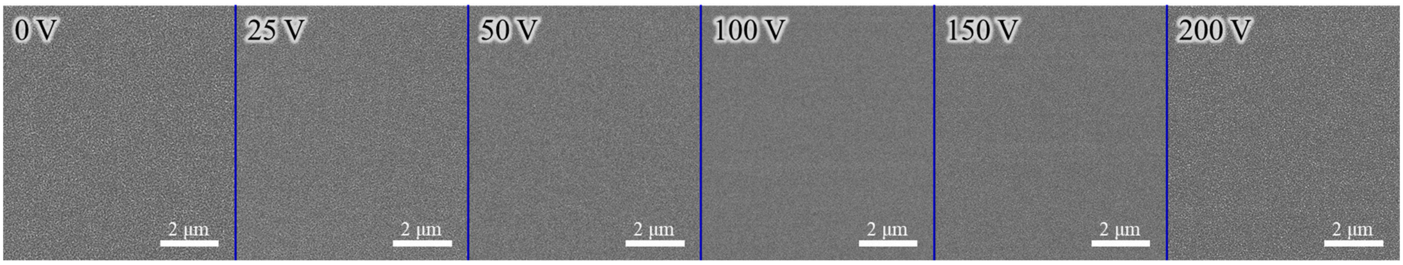

Figure S2. Low magnification SEM images as a function of substrate bias in the AlCrN coatings.

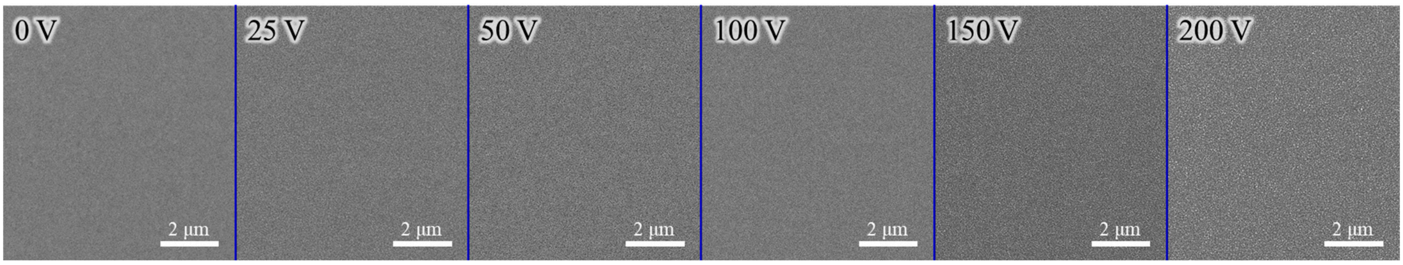

Figure S3. Low magnification SEM images as a function of substrate bias in the AlCrSiN coatings.
